# Drug-Induced Acute Myocardial Infarction: Identifying ‘Prime Suspects’ from Electronic Healthcare Records-Based Surveillance System

**DOI:** 10.1371/journal.pone.0072148

**Published:** 2013-08-28

**Authors:** Preciosa M. Coloma, Martijn J. Schuemie, Gianluca Trifirò, Laura Furlong, Erik van Mulligen, Anna Bauer-Mehren, Paul Avillach, Jan Kors, Ferran Sanz, Jordi Mestres, José Luis Oliveira, Scott Boyer, Ernst Ahlberg Helgee, Mariam Molokhia, Justin Matthews, David Prieto-Merino, Rosa Gini, Ron Herings, Giampiero Mazzaglia, Gino Picelli, Lorenza Scotti, Lars Pedersen, Johan van der Lei, Miriam Sturkenboom, on behalf of the EU-ADR consortium

**Affiliations:** 1 Department of Medical Informatics, Erasmus MC University Medical Center, Rotterdam, The Netherlands; 2 Department of Clinical and Experimental Medicine and Pharmacology, Section of Pharmacology, University of Messina, Messina, Italy; 3 Research Programme on Biomedical Informatics (GRIB), IMIM-Hospital del Mar - Universitat Pompeu Fabra, Barcelona, Spain; 4 Laboratoire d’Epidémiologie, de Statistique et d’Informatique Médicales (LESIM), L’Institut de Santé Publique, d’Épidémiologie et de Développement (ISPED), University Bordeaux Segalen, Bordeaux, France; 5 Laboratoire d’Enseignement et de Recherche sur le Traitement de l’Information Médicale (LERTIM), Faculté de Médecine, Université Aix Marseille 2, Marseille, France; 6 Instituto de Engenharia Electrónica e Telemática de Aveiro (IEETA), Universidade de Aveiro, Aveiro, Portugal; 7 Safety Assessment, AstraZeneca Research and Development, Mölndal, Sweden; 8 Department of Primary Care and Public Health Sciences, Kings College London, London, United Kingdom; 9 Faculty of Epidemiology and Population Health, London School of Hygiene and Tropical Medicine, London, United Kingdom; 10 Agenzia Regionale di Sanità della Toscana, Florence, Italy; 11 PHARMO Institute, Utrecht, The Netherlands; 12 Società Italiana di Medicina Generale, Florence, Italy; 13 Pedianet-Società Servizi Telematici SRL, Padova, Italy; 14 Department of Statistics, Università di Milano-Bicocca, Milan, Italy; 15 Department of Clinical Epidemiology, Aarhus University Hospital, Aarhus, Denmark; 16 Department of Epidemiology, Erasmus MC University Medical Center, Rotterdam, The Netherlands; Indian Institute of Toxicology Research, India

## Abstract

**Background:**

Drug-related adverse events remain an important cause of morbidity and mortality and impose huge burden on healthcare costs. Routinely collected electronic healthcare data give a good snapshot of how drugs are being used in ‘real-world’ settings.

**Objective:**

To describe a strategy that identifies potentially drug-induced acute myocardial infarction (AMI) from a large international healthcare data network.

**Methods:**

Post-marketing safety surveillance was conducted in seven population-based healthcare databases in three countries (Denmark, Italy, and the Netherlands) using anonymised demographic, clinical, and prescription/dispensing data representing 21,171,291 individuals with 154,474,063 person-years of follow-up in the period 1996–2010. Primary care physicians’ medical records and administrative claims containing reimbursements for filled prescriptions, laboratory tests, and hospitalisations were evaluated using a three-tier triage system of detection, filtering, and substantiation that generated a list of drugs potentially associated with AMI. Outcome of interest was statistically significant increased risk of AMI during drug exposure that has not been previously described in current literature and is biologically plausible.

**Results:**

Overall, 163 drugs were identified to be associated with increased risk of AMI during preliminary screening. Of these, 124 drugs were eliminated after adjustment for possible bias and confounding. With subsequent application of criteria for novelty and biological plausibility, association with AMI remained for nine drugs (‘prime suspects’): azithromycin; erythromycin; roxithromycin; metoclopramide; cisapride; domperidone; betamethasone; fluconazole; and megestrol acetate.

**Limitations:**

Although global health status, co-morbidities, and time-invariant factors were adjusted for, residual confounding cannot be ruled out.

**Conclusion:**

A strategy to identify potentially drug-induced AMI from electronic healthcare data has been proposed that takes into account not only statistical association, but also public health relevance, novelty, and biological plausibility. Although this strategy needs to be further evaluated using other healthcare data sources, the list of ‘prime suspects’ makes a good starting point for further clinical, laboratory, and epidemiologic investigation.

## Introduction

Drug-related adverse events remain an important cause of morbidity and mortality and impose a burden on healthcare costs. [1], [2], [3] There is continuous influx of new drugs into the worldwide market, but pre-approval clinical trials are unable to detect rare adverse events and to provide a complete picture of a drug’s safety profile, which evolves over its lifetime on the market. [4], [5], [6] Once a drug is made available outside the limited study population of clinical trials, there are bound to be changes in the circumstances of the drug’s actual clinical use (including exposure of broader population than was included in the clinical trials, off-label indications, concomitant use with other drugs, and dosing regimen changes) which may give rise to previously unobserved adverse effects. Post-marketing surveillance has traditionally been carried out by systematic manual review of spontaneous reports of adverse drug reactions (ADRs). Enormous improvements in computing capabilities have provided opportunities to partially automate detection of potentially drug-induced adverse events and various international initiatives are exploring new approaches to do this, primarily through data mining of electronic healthcare records. [7], [8], [9].

Electronic healthcare data, collected in the course of actual clinical practice by physicians or of healthcare utilisation by insurers and health maintenance organisations, give a good snapshot of how drugs are being used in ‘real-world’ settings. Being routine by-products of the healthcare delivery system, the use of such data offers the advantage of efficiency in terms of time, manpower, and financial costs needed to investigate patient safety issues. While the advantages of automated surveillance are obvious, there are growing concerns that such data mining may generate more signals than can be followed up effectively with currently available resources. This concern is not entirely unfounded, considering that the annual volume of reports received in spontaneous reporting systems (SRS), database systems primarily designed for signal detection, has become enormous and unmanageable. [10], [11] The problem is likely to be worse with the use of EHR data which have been intended for other purposes and which can be mined for associations without routine human evaluation of potential alternative explanations.

Detection of safety signals is only the initial step in the long and complex process of post-marketing safety surveillance. The evaluation of a signal may take years, from the earliest suspicion of a potential risk to an established mechanism of causation and fully understood phenomenon. [12] While signals derived from EHR data may give a good snapshot of how drugs are being used in real-world settings, there remains the need to establish guidelines as to when - and how - to consider a safety signal likely to be substantial enough to warrant verification and follow-up. Various strategies for signal prioritisation have been proposed in many publications, although most of these refer to signals derived from SRS. [12], [13], [14], [15], [16] These strategies focus consistently on signals with serious adverse effect, strong supporting evidence, and greatest public health impact.

In this paper we describe findings from post-marketing surveillance using healthcare data of over 20 million individuals from three European countries within the EU-ADR network (http:\www.euadr-project.org). We look at primary care physicians’ medical records which comprise detailed clinical information including patients’ symptoms, physical examination findings, diagnostic test results, and prescribed medications or other interventions. We also look at administrative claims that document reimbursements for filled prescriptions, laboratory and ancillary tests, as well as hospitalisations. Taking the adverse event acute myocardial infarction (AMI) as an example, we describe a strategy for combining evidence from different data sources to identify associations that may represent genuine risk and, hence, necessitate further investigation through formal hypothesis testing studies or action from drug regulatory agencies.

## Methods

### Data Sources

Identification of ‘prime suspects’ was performed in seven databases of the EU-ADR network [8] for the period 1996–2010: (1) Health Search/CSD LPD (HSD, Italy); (2) Interdisciplinary Processing of Clinical Information (IPCI, Netherlands); (3) Pedianet (Italy); (4) PHARMO Network (PHARMO, Netherlands); (5) Aarhus University Hospital Database (Aarhus, Denmark); (6) Lombardy database (Lombardy, Italy); and (7) Tuscany database (Tuscany, Italy). HSD, IPCI, and Pedianet are primary care/general practitioner (GP) databases, where clinical information and drug prescriptions are recorded. Aarhus, PHARMO, Lombardy, and Tuscany are comprehensive record-linkage systems where drug dispensing data are linked to registries containing hospitalisation and other services. **Table 1** provides an overview of the characteristics of each database. All of the databases in EU-ADR have been widely used for pharmacoepidemiologic research, have well-developed safeguard mechanisms ensuring patient data protection, and have been validated for a variety of drug exposures and clinical outcomes. [17], [18], [19], [20], [21], [22], [23] Most healthcare services, including pharmaceutical services, are provided for, or subsidised by, the state in Italy and Denmark and covered by obligatory health insurance in the Netherlands and turnover is low. In all of the countries with GP databases, GPs function as ‘gatekeepers’ of the healthcare system. A more detailed description of the database network can be found in earlier publications. [8], [24] Healthcare data used in this study represent anonymised demographic and healthcare information from 21,171,291 individuals with 154,474,063 person-years of follow-up.

**Table 1:** Characteristics of the databases in the EU-ADR network.

CHARACTERISTICS	Pedianet (Italy)	HSD (Italy)	Lombardy Regional (Italy)	Tuscany Regional (Italy)	IPCI (Netherlands)	PHARMO (Netherlands)	QRESEARCH* (UK)	Aarhus (Denmark)
**Current source population**	160,000 children	1,500,000	9,000,000	3,500,000	1,500,000	3,000,000	4,000,000	1,800,000
**Years covered for this study**	2003–2007	2003–2007	2003–2005	2003–2006	1996–2006	1998–2007	2000–2007	2001–2006
**Type of database**	General Practice pediatric database	General Practice database	Administrative	Administrative	General Practice database	Hybrid(administrativeand medicalrecord/registries)	General Practice database	Administrative
**Age range**	0–14	From 15 onwards	All ages	All ages	All ages	All ages	All ages	All ages
**% Males**	52.2	47.2	48.8	48.1	49.6	45.8	49.6	49.9
**Demographic information available**								
Date of registration	Yes	Yes	Yes	Yes	Yes	Yes	Yes	Yes
Date of transferring out	Yes	Yes	Yes	Yes	Yes	Yes	Yes	Yes
Date of birth	MM-YY	MM-YY	DD-MM-YY	DD-MM-YY	MM-YY	DD-MM-YY	YY	MM-YY
Gender	Yes	Yes	Yes	Yes	Yes	Yes	Yes	Yes
Ethnicity/Race	No	No	No	No	No	No	No	No
**Drug information available**								
Product coding	MINSAN	MINSAN	MINSAN	MINSAN	HPK	Z index	EMIS	VAerets
Active international principlecoding system	ATC	ATC	ATC	ATC	ATC	ATC	BNF	ATC
Date of prescription/dispensing	Yes	Yes	Yes	Yes	Yes	Yes	Yes	Yes
Dosing regimen	Yes	Yes	No	No	Yes	Yes	Yes	Yes
Quantity	Yes	Yes	Yes	Yes	Yes	Yes	Yes	Yes
Indication of use	Yes	Yes	No	No	Yes	Yes for in-hospital	No	Yes
**Outcome information available**								
Symptoms (Yes/No)	Yes, as free text/codes	Yes, as freetext/codes	No	No	Yes, as freetext/codes	Yes for some	Yes, as codes	No
Outpatient primarycare diagnoses	Yes, as free text/codes	Yes Freetext/codes	No	No	Yes, as freetext/codes	No	Yes	No
Outpatient specialistcare diagnoses	Yes, as free text/codes	Yes	No	No	Yes	No	Yes	No
Hospital discharge diagnoses	Yes, as free text/codes	Yes, as freetext/codes	Yes	Yes	Yes, as freetext/codes	Yes	Yes	Yes
Diagnosis coding scheme	ICD-9CM	ICD-9CM	ICD-9CM	ICD-9CM	ICPC	ICD-9CM	RCD	ICD-10
Diagnostic procedures	Yes	Yes	Yes	Yes	No	Yes for in-hospital interventions	Yes	Yes, in-hospital only
Laboratory tests	Yes	Yes	No	No	Yes	Yes subset	Yes	Yes, in-hospital only

**Legend:**

**ICPC**: International Classification of Primary Care.

**ICD9-CM**: International Classification of Diseases –9th revision Clinical Modification.

**RCD**: READ CODE Classification.

**ICD-10**: International Classification of Diseases –10th revision.

**MINSAN**: Italian Ministry of Health.

**NOTE**: * QRESEARCH did not contribute data for the analyses described in this paper.

### Ethical Approval

The respective Scientific and Ethics committees of each database approved the use of the data for this study. All of the databases in the EU-ADR network adhere to local governance rules regarding the storage of patient data and its use for research and have well-developed safeguard mechanisms ensuring compliance with the European directives and national regulations; no individual written informed consent was required for this study.

### Distributed Data Processing

A distributed database network approach was chosen in EU-ADR, allowing database custodians to maintain local control of their data, while reaching the goal of sharing data in a standardised manner. Input data files are created locally and are subsequently managed by purpose-built software called Jerboa^©^, written entirely in Java™ to ensure that it will run in a wide variety of computational environments. The software queries patient-level data in the different databases, which are later aggregated, de-identified and sent in encrypted format to a central repository for evaluation and further analysis. This repository is managed by the Department of Medical Informatics at Erasmus Medical Center in the Netherlands, the project’s coordinating centre.

### Identification and Validation of Cases of Acute Myocardial Infarction

Each of the databases in the EU-ADR network has unique characteristics depending on its primary objective and local function (i.e. administrative claims or medical records) and contains medical information coded according to different (natural) languages and disease terminologies. Potential cases of AMI were identified using search queries that utilised three disease coding terminologies: (1) International Classification of Primary Care (ICPC) for IPCI; (2) International Classification of Diseases 9th revision-Clinical Modification (ICD-9CM) for ARS, HSD, Lombardy, and PHARMO; and (3) ICD-10th revision for Aarhus. To extract the same event across databases, these different terminologies were mapped using the Unified Medical Language System, a biomedical terminology integration system handling more than 150 terminologies. The mapping ensured that AMI was described using a common language. We identified AMI from the databases using an iterative process that included harmonising definitions based on clinical criteria established from literature, using diagnosis codes and free text as well as laboratory findings when available. We inspected differences in event ascertainment by comparing data queries and benchmarking age-specific and standardised incidence rates of the events (direct standardisation was carried out using the WHO World Standard Population). The incidence rates we obtained in EU-ADR are consistent with what has been cited in previous literature. The multi-step process of terminology mapping, harmonisation and benchmarking for the data extraction for AMI (and for four other events) has been described in more detail in earlier publications. [25], [26] We reproduce in **Figure S1** (available as supplementary file online) the schematic diagram summarising the harmonisation process of event identification across the databases in EU-ADR.

Case validation by manual review of hospitalisation records and GP records was done in a random subset of the cases. The overall positive predictive value (PPV) for identifying AMI was good, ranging from 75% (ICPC) to 95% (ICD9-CM) to 100% (ICD-10). These findings are consistent with PPV estimates for ICD9-CM and ICD-10 cited in the literature (To date there is no study describing the PPV of ICPC codes or free text search for identifying AMI). [27].

Only the first occurrence of AMI (i.e. incident case) was considered in the analyses; patient time after an AMI was censored.

### Drug Exposure

Drug prescription/dispensing data were used to estimate event rates during drug exposure and were assessed according to the Anatomical Therapeutic Chemical (ATC) classification system of the World Health Organization (WHO) (http://www.whocc.no/atc/structure_and_principles/). The duration covered by each prescription or dispensing was estimated according to legend duration (if dosing regimen is available), or otherwise based on the defined daily dose (http://www.whocc.no/ddd/definition_and_general_considera/). Overlapping treatment episodes with the same drug were combined into a single episode of drug use that starts when the first prescription begins and stops when the last prescription ends. When a patient uses more than one drug at a time, the corresponding person-time is labelled accordingly. Events are assigned to the episodes (drug use/non-use) in which they occurred.

### Screening for ‘Prime Suspects’

**Figure 1:**
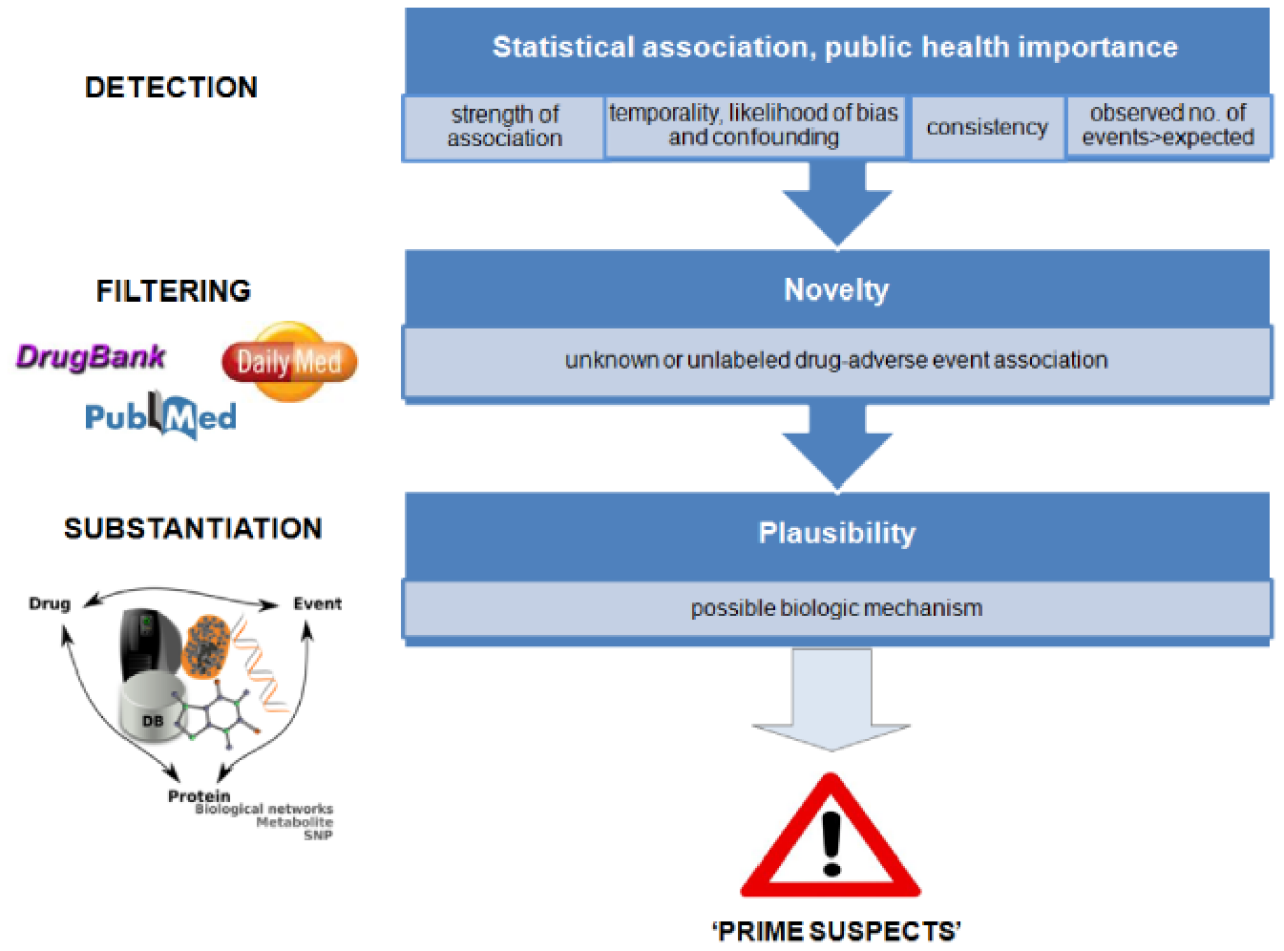
Three-tier triage system (detection, filtering, and substantiation) for detecting ‘prime suspects’.

We developed a three-tier triage system (detection, filtering, and substantiation) that generated a list of drugs potentially associated with AMI (**Figure 1**).

#### Strength of statistical association

In the EU-ADR Project we have applied a wide range of statistical methods, including case-based methods (e.g., case control and self-controlled case series), cohort methods, as well as methods developed initially for use in spontaneous ADR reporting systems. We have previously evaluated the relative performance of these methods for detecting known ADRs from EHR data and our findings showed that combinations of methods demonstrate good performance in distinguishing known ADRs from negative controls. [28] Among these methods, the Longitudinal Gamma Poisson Shrinker (LGPS, an adaptation of the GPS, a data mining technique widely used in spontaneous reporting systems to detect potential ADRs) [29] was the best-performing among the methods. We calculated the relative risk, RR_LGPS_ and used this to rank the initial list of ‘prime suspects.’ The results from the different databases were combined to generate a single risk estimate per drug as if the databases together form one large database. We did not perform any meta-analyses. A value of RR_LGPS_≥2.0 and a lower 95% CI of RR_LGPS_>1 were used as threshold values for further processing. A more detailed description of LGPS and how the RR_LGPS_ is calculated is given in **Appendix S1** (available as supplementary file online).

#### Alternative explanations for the identified associations: protopathic bias and confounding

Another method, LEOPARD (Longitudinal Evaluation of Observational Profiles of Adverse events Related to Drugs), developed in EU-ADR, attempts to single out associations that may be detected because the drug is used to treat the event, or a prodrome of the event, rather than cause it (protopathic bias). [29] For every suspect drug, LEOPARD compares the rates of prescription starts within a fixed window (±25 days) before and after the event. An increase in the number of prescriptions after an event relative to number of prescriptions before the event is taken to be an indication of protopathic bias. All drug-related AMI flagged by LEOPARD as possibly due to protopathic bias were eliminated from the list. To account for possible confounding, we further sorted out the list and considered only associations that had significant increased risk estimates based on the matched case-control method (lower 95% CI of exposure odds ratio (OR)>1) or the self-controlled case series (SCCS) (lower 95% CI of incidence rate ratio (IRR_SCCS_)>1). In the case control method, each case was matched to two controls of same age, sex, and index date (i.e. date of AMI). To adjust for co-morbidity and global patient health status, we used as proxy the number of different drugs an individual was exposed to within the period one year and one month prior to index date. We also employed the SCCS method which controls for time-fixed confounders such as genetic factors, socio-economic status, individual frailty, and severity of underlying disease.

#### Public health importance

To quantify the public health impact of potentially drug-induced AMI, we used as surrogate the number of excess cases of patients exposed to the drug relative to the background unexposed population (observed – expected).

### Automated Filtering and Substantiation of Signals

We have developed in the EU-ADR Project a web-based platform that allows systematic analysis of potential safety signals through several distributed software, streamlined into a single computational workflow (https://bioinformatics.ua.pt/euadr). The entry point of the system is a potential drug safety signal, which is composed of a drug and its associated adverse event (in this case AMI). Both signal filtering and substantiation are carried out using dedicated bioinformatics methods integrated into processing pipelines by means of Taverna, an open source workflow management system used to design and execute scientific workflows and aid *in silico* experimentation. We provide in **Figure S2** (available as supplementary file online) a schematic representation of the web platform set up. A more comprehensive description of the EU-ADR web platform can be found in other publications. [30], [31].

#### Novel associations

The interest in drug safety surveillance is discovery of phenomenon describing a ‘new potentially causal association, or a new aspect of a known association.’ [32] To discriminate among potentially relevant new and already known associations, we used the abovementioned web platform to assess previous reporting of such drugs with AMI in the biomedical literature and eliminated from the list of ‘prime suspects’ drugs previously reported to be associated with AMI in more than one of three biomedical databases: MEDLINE (http://www.ncbi.nlm.nih.gov/pubmed); DrugBank (http://www.drugbank.ca/); or DailyMed (http://dailymed.nlm.nih.gov/dailymed/about.cfm). The Medline ADR signal filtering workflow automates literature analysis by assessing a list of publications regarding AMI. The algorithm adopts a semantics-based approach that processes Medline annotations looking for particular MeSH terms. This workflow’s output is a direct relationship between AMI and its descriptions in Medline, if present. In addition, there is a signal filtering that identifies co-occurrence of the drug and the event (in this case AMI) in Medline literature (Medline Co-occurrence) or drug databases such as DailyMed (http://dailymed.nlm.nih.gov/) or DrugBank (http://www.drugbank.ca/). The workflows use statistical and text-mining techniques to evaluate drug names, ATC codes and AMI co-occurrences in the indexed resources.

#### Substantiation for biological plausibility

**Figure 2:**
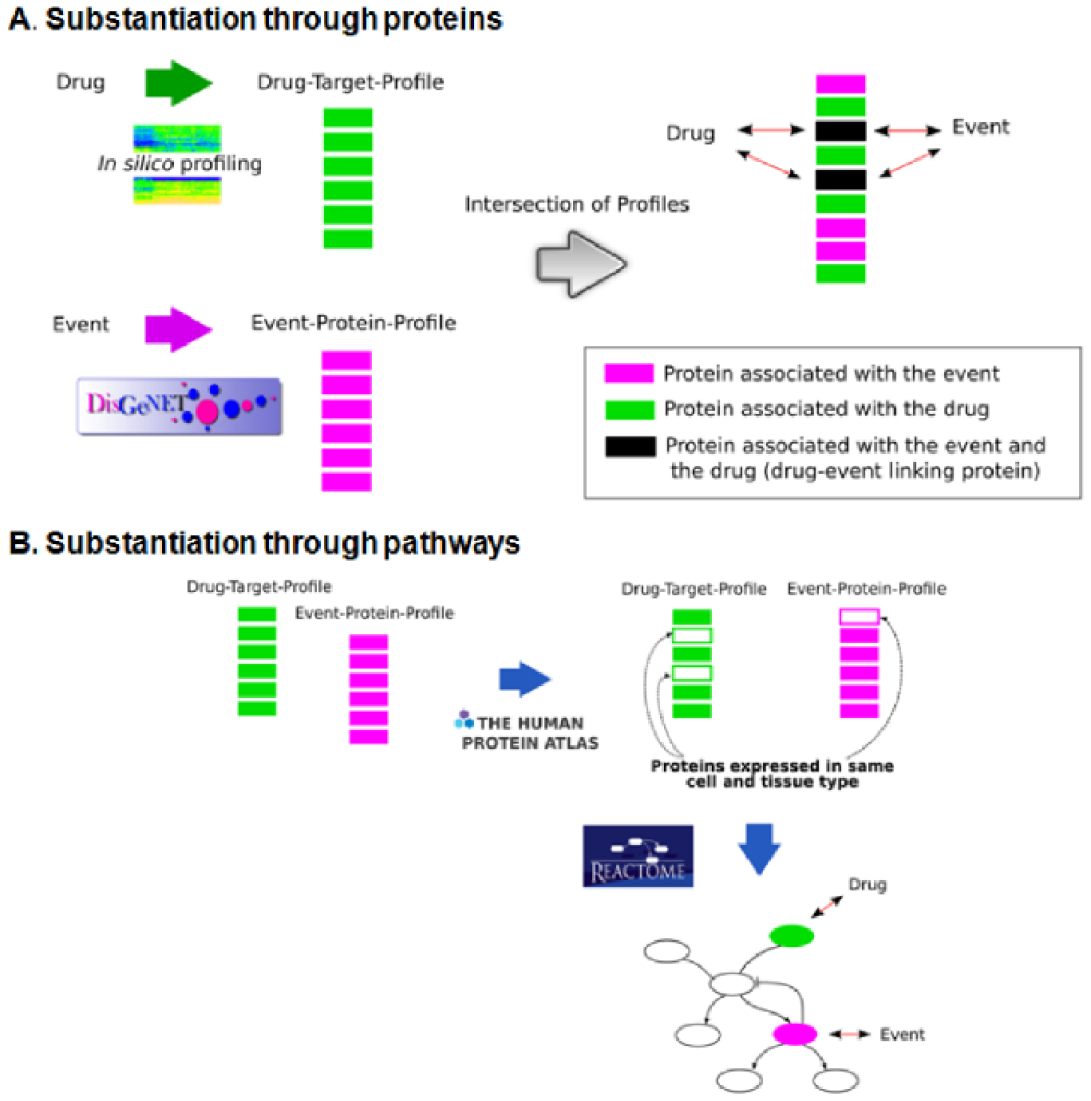
Schematic representation of the process of substantiation of suspected drug-induced adverse events via proteins (A) and via pathways (B). (From Bauer-Mehren A, van Mulligen EM, Avillach P, Carrascosa Mdel C, Garcia-Serna R, et al. (2012) Automatic filtering and substantiation of drug safety signals. *PLoS Comput Biol 8: e1002457*. Reproduced with permission from the authors).

As a final assessment procedure, we retained in the ‘prime suspects’ list only those associations for which a possible biologic mechanism could be found. Automatic linkage of biomedical entities (drugs, proteins and their genetic variants, biological pathways and clinical events) via customised bioinformatics methods was done to find supporting evidence for the ‘prime suspects’ (see **Figure 2**). The associations that passed the screening described above were processed by a computational framework that identifies pair-wise relationships between the drug and AMI based on *in silico* prediction of drug targets, analysis of drug metabolites and gene-disease associations. [30].

Using the above substantiation requirement may preclude finding drugs that induce AMI with mechanisms that cannot be predicted from the drug’s pharmacological action. To account for this type of ADRs, we determined which drugs would remain if we keep those for which the substantiation workflow did not find anything, but passed the novelty requirement. A manual literature search was further performed to determine a logical explanation for these associations.

## Results

### Identifying ‘Prime Suspects’

Overall, we found 235,283 cases of AMI (both drug-related and non drug-related) during the period 1996–2010, with a background incidence rate of 153.7 per 100,000 person-years. We initially identified 163 drugs possibly associated with AMI. We subsequently flagged, and discarded from the list, 72 drugs as likely being used to treat prodromal symptoms of AMI rather than cause it (i.e. due to protopathic bias). Systemic antibiotics comprised about one-fourth of the suspect drugs (22 drugs out of 91), with the rest involving 14 other therapeutic classes. Adjustment for confounding reduced the number of suspect drugs to 39. The number of excess cases attributable to drug exposure ranged from 18 (for the antibiotic rokitamycin) to 2,445 (for metformin fixed-dose combinations). **Table 2** shows the list of suspect drugs that passed preliminary screening, ranked according to a surrogate of public health importance: the number of excess cases.

**Table 2:** Drugs potentially associated with acute myocardial infarction^†^.

Therapeutic class	Drug	RR_LGPS_(*95% CI*)	OR(*95% CI*)	IRR_SCCS_(*95% CI*)	No. ofexcess cases
Oral hypoglycemic agent	Metformin and sulfonamides	2.5 (*2.4, 2.6*)	1.9 (*1.8, 2.0*)	1.5 (*1.4, 1.6*)	2,445
Antihypertensive	Nifedipine	2.1 (*2.0, 2.2*)	1.6 (*1.6, 1.7*)	1.8 (*1.7, 2.0*)	2,097
Systemic corticosteroid	Prednisone	2.5 (*2.4, 2.6*)	1.5 (*1.4, 1.6*)	2.2 (*1.9, 2.6*)	1,261
β-adrenergic agonist	Salbutamol (systemic)	2.1 (*2.0, 2.2*)	1.2 (*1.2, 1.3*)	1.9 (*1.6, 2.2*)	1,017
Systemic corticosteroid	Methylprednisolone	2.3 (*2.2, 2.4*)	1.5 (*1.3, 1.6*)	2.0 (*1.7, 2.3*)	832
Opioid analgesic	Tramadol	2.1 (*2.0, 2.2*)	1.3 (*1.2, 1.4*)	2.2 (*1.7, 2.8*)	736
Oral hypoglycemic agent	Glibenclamide	2.2 (*2.1, 2.4*)	1.6 (*1.6, 1.8*)	1.3 (*1.1, 1.6*)	686
Antihypertensive	Clonidine	2.9 (*2.7, 3.1*)	1.8 (*1.6, 1.9*)	2.5 (*1.9, 3.2*)	650
Systemic antibiotic	Clarithromycin	3.5 (*3.2, 3.7*)	2.4 (*2.2, 2.6*)	3.3 (*2.8, 3.8*)	645
β-adrenergic agonist	Fenoterol (inhaled)	2.5 (*2.3, 2.6*)	1.4 (*1.3, 1.5*)	1.6 (*1.1, 2.3*)	588
β-adrenergic agonist	Salbutamol (inhaled)	2.4 (*2.2, 2.6*)	1.3 (*1.2, 1.4*)	1.7 (*1.4, 2.2*)	510
Systemic antibiotic	Amoxicillin	2.2 (*2.0, 2.3*)	1.6 (*1.5, 1.8*)	2.0 (*1.8, 2.4*)	497
Systemic corticosteroid	Betamethasone	2.9 (*2.7, 3.2*)	1.7 (*1.5, 2.0*)	3.3 (*2.6, 4.3*)	365
Antacid	Magaldrate	2.8 (*2.5, 3.0*)	1.9 (*1.7, 2.2*)	4.8 (*3.9, 5.9*)	365
Systemic antibiotic	Phenoxymethylpenicillin	3.6 (*3.3, 4.0*)	2.6 (*2.3, 2.9*)	3.8 (*3.0, 4.9*)	335
Systemic corticosteroid	Dexamethasone	3.2 (*2.9, 3.5*)	1.9 (*1.7, 2.2*)	5.4 (*4.1, 7.2*)	285
Antacid	Combinations of aluminum,magnesium, or calcium salts	3.1 (*2.8, 3.5*)	1.9 (*1.6, 2.2*)	4.4 (*3.3, 5.7*)	265
Opioid analgesic	Fentanyl	2.5 (*2.3, 2.8*)	1.2 (*1.1, 1.4*)	2.1 (*1.2, 3.9*)	249
Antiemetic/gastric prokinetic	Metoclopramide	5.7 (*5.1, 6.4*)	2.6 (*2.2, 3.1*)	8.9 (*5.1, 15.6*)	236
Antiemetic/gastric prokinetic	Domperidone	2.8 (*2.5,3.1*)	1.6 (*1.4, 1.8*)	3.1 (*2.4, 4.0*)	229
Systemic antibiotic	Azithromycin	2.8 (*2.5, 3.2*)	1.7 (*1.5, 2.1*)	2.5 (*1.8, 3.5*)	159
Systemic antibiotic	Pivampicillin	4.5 (*3.9, 5.2*)	3.1 (*2.6, 3.7*)	3.6 (*2.1, 6.1*)	156
Systemic antibiotic	Ceftriaxone	8.2 (*7.0, 9.4*)	5.2 (*2.1, 13.1*)	5.6 (*2.8, 11.0*)	154
Nonsteroidalanti-inflammatory drug	Ketorolac	4.6 (*3.9, 5.3*)	2.7 (*2.1, 3.4*)	2.8 (*1.7, 4.7*)	135
Other anti-anemic	Darbepoetin alfa	3.3 (*2.8, 3.8*)	1.7 (*1.4, 2.1*)	3.2 (*1.8, 5.6*)	126
Systemic antibiotic	Cefixime	3.1 (*2.6, 3.6*)	2.4 (*1.9, 3.1*)	4.4 (*3.1, 6.2*)	104
Systemic antibiotic	Roxithromycin	3.3 (*2.8, 3.9*)	2.4 (*1.9, 3.0*)	2.9 (*1.8, 4.9*)	89
Opioid analgesic	Ketobemidone and antispasmodics	2.2 (*1.9, 2.6*)	1.2 (*1.0, 1.5*)	2.6 (*1.2, 5.7*)	78
Systemic antibiotic	Dicloxacillin	2.6 (*2.2, 3.1*)	1.8 (*1.5, 2.2*)	2.5 (*1.2, 5.2*)	73
Antiemetic/gastric prokinetic	Cisapride	2.1 (*1.8, 2.5*)	1.2 (*1.0, 1.5*)	2.4 (*1.6, 3.6*)	69
Antineoplastic/immunomodulator	Azathioprine	2.1 (*1.7, 2.4*)	1.2 (*1.1, 1.5*)	3.4 (*1.9, 6.1*)	69
Oral hypoglycemic agent	Gliquidone	2.7 (*2.2, 3.2*)	2.1 (*1.6, 2.7*)	2.2 (*1.2, 4.0*)	66
Systemic antibiotic	Erythromycin	3.7 (*3.0, 4.6*)	2.6 (*1.9, 3.4*)	2.4 (*1.1, 5.1*)	63
Systemic antifungal	Fluconazole	2.7 (*2.2, 3.3*)	1.5 (*1.2, 2.0*)	2.2 (*1.2, 4.4*)	53
Phosphate binder	Polystyrene sulfonate	4.8 (*3.6, 6.4*)	2.2 (*1.5, 3.1*)	3.3 (*1.1, 10.3*)	48
Antiemetic/gastric prokinetic	Butylscopolamine	5.8 (*4.2, 7.7*)	2.1 (*1.4, 3.4*)	11.3 (*5.0, 25.8*)	45
Antineoplastic/immunomodulator	Megestrol	3.2 (*2.5, 4.0*)	2.5 (*1.8, 3.4*)	4.0 (*1.8, 9.3*)	44
Systemic antibiotic	Ceftibuten	2.3 (*1.8, 3.0*)	1.9 (*1.3, 2.7*)	3.0 (*1.7, 5.2*)	31
Systemic antibiotic	Rokitamycin	2.6 (*1.8, 3.7*)	1.8 (*1.1, 3.0*)	4.3 (*2.3, 8.0*)	18

### Filtering and Substantiation to Determine Novelty and Plausibility of Associations

Out of the 39 drugs that passed initial screening, only 11 are previously known from literature to be associated with AMI. After applying criteria for both novelty and plausibility, we arrived at nine ‘prime suspects’: the systemic macrolide antibiotics erythromycin roxythromycin, and azithromycin; the gastric prokinetic agents metoclopramide, cisapride, and domperidone; the antifungal fluconazole; and the steroidal drugs betamethasone and megestrol acetate (see **Table 3**).

**Table 3:** Table 3. ‘Prime suspects’: drugs potentially associated with increased risk of acute myocardial infarction which passed the filtering (i.e. novelty) and substantiation (i.e. biological plausibility) criteria.

Drugs that satisfied both novelty and plausibility criteria	Drugs that satisfied only novelty criterion
**Metoclopramide**	Combinations of aluminum, magnesium, and calcium salts
**Cisapride**	Magaldrate
**Domperidone**	Butylscopolamine
**Betamethasone**	Gliquidone
**Erythromycin**	Metformin combinations with sulfonamides
**Roxithromycin**	Methylprednisolone
**Azithromycin**	Pivampicillin
**Fluconazole**	Phenoxymethylpenicillin
**Megestrol acetate**	Dicloxacillin
	Ceftriaxone
	Cefixime
	Ceftibuten
	Rokitamycin
	Azathioprine
	Ketobemidone and antispasmodics
	Fenoterol (inhaled)
	Salbutamol (inhaled)
	Polystyrene sulfonate

### Second Look at ‘Prime Suspects’: Idiosyncratic Reactions

Consideration of associations not substantiated by a known biologic mechanism increased the number of ‘prime suspects’ to 27 (**Table 3**). Butylscopolamine is another prokinetic drug; methylprednisolone is another corticosteroid; while pivampicillin, phenoxymethylpenicillin, dicloxacillin, ceftriaxone, cefixime, ceftibuten, and rokitamycin are all β-lactam antibiotics except for the last one, which is a macrolide. Other drugs include the bronchodilators fenoterol and salbutamol, antacids, the opioid ketobemidone, and the phosphate binder polysterene sulfonate.

## Discussion

We have described a strategy that identifies and prioritises potentially drug-induced acute myocardial infarction from routinely collected healthcare data. We attempted to simulate how a physician or drug regulator would go about evaluating suspected drug-induced events. This is the first triage strategy for safety surveillance developed for use – and tested – in data from electronic healthcare records. In this strategy, we take into account public health relevance, novelty, and biological plausibility in addition to statistical association. Stepwise exclusion of alternative causes is part of an etiology-based approach for the assessment of ADRs. [33], [34] While usually inherent in physician-reported ADRs, such is not the case with associations obtained from secondary healthcare data (particularly with insurance/administrative claims), which are inferred outside the actual physician-patient encounter. We tried to offset this limitation by adjusting for bias and confounding. The mechanisms behind most ADRs are still not completely understood, but accumulating evidence over the years indicate the interplay of various factors and increasing role of inter-individual genetic variants in genes encoding drug-metabolising enzymes and drug target genes. [35] The triage strategy we developed takes into account various pathways that can lead to a plausible explanation of the identified associations.

**Figure 3:**
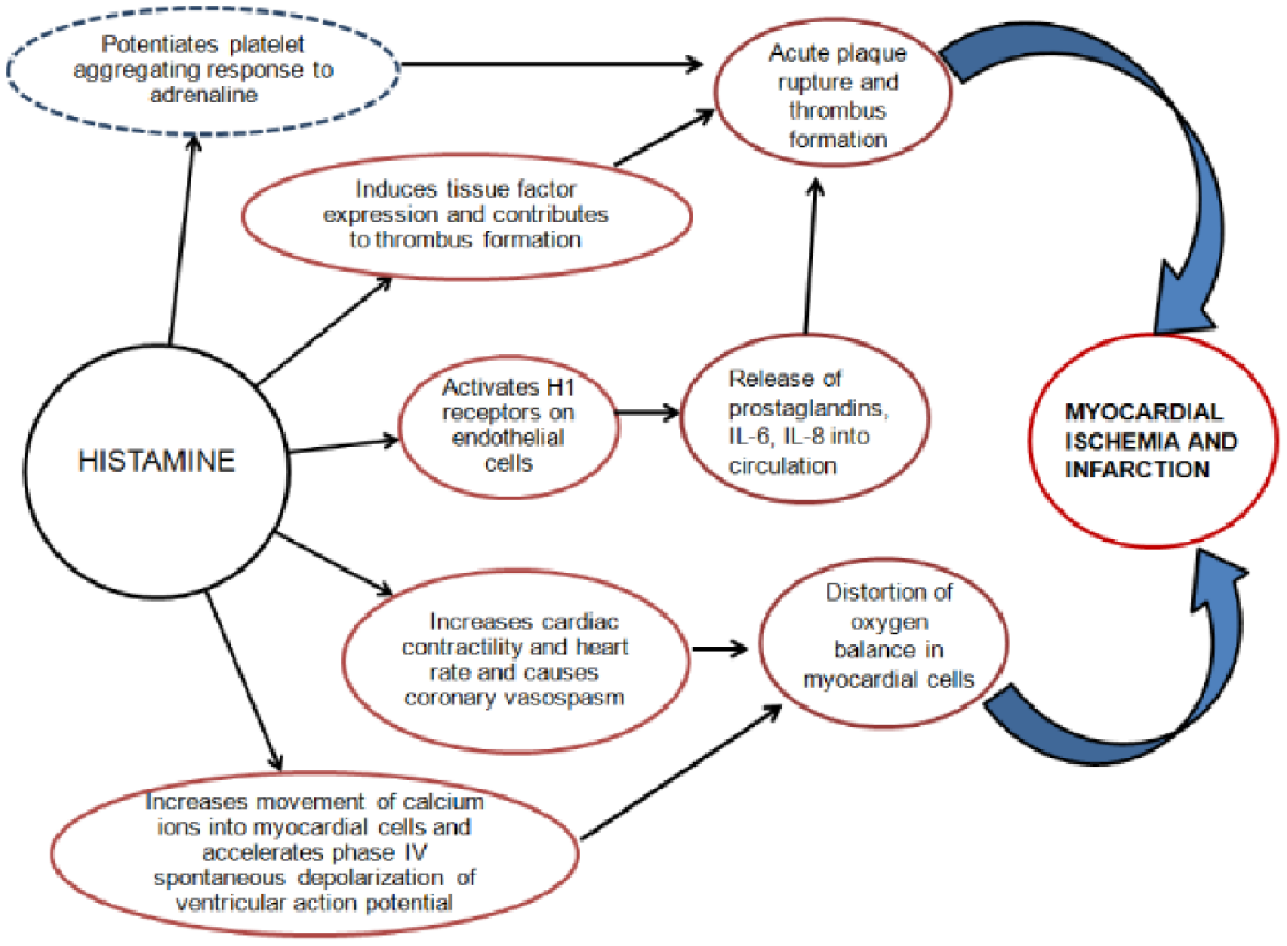
Central role of histamine in drug-induced acute myocardial infarction via Kounis syndrome. Aside from its direct vasoconstricting and thrombus-generating effects, histamine also potentiates the platelet aggregating response to adrenaline (dotted outline).

Because drugs belonging to the same class often have a similar pharmacological mechanism of therapeutic action and adverse effects, [36], [37] we assumed that associations involving drugs of the same class may require more thorough investigation: systemic antibiotics comprised about 25% of the initial list of suspect drugs. The proposed mechanism underlying this association is via allergic angina progressing to AMI. The occurrence of chest pain and allergic-anaphylactic reaction, accompanied by clinical and laboratory findings of classical angina pectoris, is caused by inflammatory mediators released during an allergic insult and constitutes the so-called Kounis syndrome. [38], [39] Several studies have shown that β-lactam antibiotics may cause allergic reactions and initiate acute coronary syndrome in hypersensitive individuals. Clinical manifestations of Kounis syndrome, including electrocardiographic findings, are similar to AMI. Kounis syndrome is largely attributed to the action of cardiac mast cells found in the coronary artery intimal layer and atherosclerotic plaques; it has been demonstrated that the density of mast cells in the culprit atheroma of patients who died from AMI was 200 times higher than the density in normal coronary vessels from the same patients. [40] These mast cells become activated during the allergic reaction and release endogenous mediators, including histamine, leukotrienes, thromboxane, platelet activation factor, tryptase, chymase, and rennin - all of which affect different receptors on the coronary vessel wall that may result in AMI. [41] Histamine, the main amine released during allergic reactions, plays a central role in the development of allergic AMI (**see** **Figure 3**). The effects of histamine on cardiac function, including increased cardiac contractility and heart rate as well as coronary vasospasm, are mediated via H1- and H2- receptors situated on the cardiac chambers and coronary arteries. In addition to direct coronary vasoconstriction and thrombus generating effects, histamine also potentiates the platelet aggregating response to adrenaline. Kounis syndrome has previously been described with use of penicillin, ampicillin, amoxicillin, cefuroxime, cefoperazone, and cefoxitin. [41] To date, there have been no reports in the literature associating macrolide antibiotics with the Kounis syndrome. It is, possible, however, that macrolides induce coronary vasopasm via the same mechanism as that of the β-lactams. [42], [43] Immediate-type hypersensitivity (i.e. anaphylaxis), non-immediate reactions like fixed drug eruptions, toxic epidermal necrolysis and leukocytoclastic vasculitis have been reported with the use of macrolides. [42], [44] Oral contraceptive use in women and recreational drug use with cocaine are the main culprits usually implicated when AMI occurs in a young patient with no clinically evident coronary artery disease (CAD) or other known cardiovascular risk factors. [45] With recent literature implicating Kounis syndrome in drug-eluting stent thrombosis, [46] there is good reason to believe that antibiotic-associated Kounis syndrome is a condition that clinicians need to be more aware of. Although the possibility of channeling bias in the association between macrolides and AMI cannot be discounted (i.e. preferential use of macrolide antibiotics in those patients who may be at higher risk for developing hypersensitivity to β-lactams and, consequently, at risk for developing Kounis syndrome), this association deserves further investigation.

Among the gastric prokinetic drugs, cisapride has the most well characterised cardiac adverse effect profile, which includes ventricular arrhythmia, QT prolongation and torsades de pointes. [47], [48], [49] Both metoclopramide and domperidone have also been reported to have arrythmogenic potential. [50] The effects of these drugs on the cardiovascular system are related to their action on dopaminergic and 5-HT receptors; this could be the same mechanism that predisposes to myocardial ischemia or infarction, although how this may happen is yet unclear. [51].

Long-term use of some drugs may increase risk for AMI by accelerating development of atherosclerosis and CAD. Any drug that alters the modifiable risk factors for CAD (e.g., cigarette smoking, elevated plasma low-density lipoprotein cholesterol, reduced plasma high-density lipoprotein cholesterol, hypertension, obesity, and diabetes) [52] has the potential to increase the risk of AMI. Lipodystrophy, weight gain, and hypertension are known corticosteroid-induced adverse effects. [53] Hyperlipidemia is usually associated with long-term corticosteroid use and cases of AMI with use of systemic corticosteroids have also been documented. [54] In a Danish study of patients with out-of-hospital cardiac arrest, use of corticosteroids, bronchodilators, and antipsychotics were found to have the strongest association up to 30 days before the event. [55] Moreover, corticosteroids are used in patients with systemic lupus erythematosus (SLE), psoriasis, and other rheumatologic diseases - accelerated atherosclerosis and premature CAD are recognised complications of these disorders, although the exact etiology remains unclear and is likely to be multifactorial. [56], [57] Megestrol acetate, a progesterone derivative used for hot flushes and for palliative treatment of hormone-dependent malignant neoplasms, may predispose to AMI via its effects on known cardiovascular risk factors: weight gain, hypertension, and hyperglycemia or diabetes mellitus occur with use of megestrol via glucocorticoid action-mediated increased peripheral insulin resistance, especially with long-term use. [58], [59], [60] Fluconazole has been associated with cardiac adverse effects including QT prolongation and torsades de pointes, [61], [62] but not with myocardial ischemia or infarction. Another drug belonging to the same class, itraconazole, has been described as causing a negative inotropic effect resulting in hypertension, hypokalemia, and edema (congestive heart failure). [63], [64] The product label of itraconazole has been changed to include a warning to avoid administration to patients with evidence, or history, of heart failure (http://dailymed.nlm.nih.gov/dailymed/lookup.cfm?setid=a4d555fa-787c-40fb-bb7d-b0d4f7318fd0). Azole antifungals may trigger AMI in those already at risk by modifying lipid profile, an important determinant of cardiovascular risk. The product label of fluconazole indicates that there have been post-marketing reports of both hypercholesterolemia and hypertriglyceridemia with fluconazole use (http://dailymed.nlm.nih.gov/dailymed/lookup.cfm?setid=f694c617-3383-416c-91b6-b94fda371204). Drug-drug interactions may also play a role in the development of AMI, especially in high-risk patients who are taking multiple cardiac drugs: all the azole antifungals inhibit CYP450 enzymes to some degree and may predispose to adverse cardiac complications, including rhythm problems and ischemia or infarction. [65], [66].

There are many recognised ADRs which cannot be predicted from a drug’s pharmacological action and whose mechanisms remain unclear and have yet to be elucidated. [67], [68] We looked at novel associations which were not obviously explained by the drug’s pharmacology. Doing away with the substantiation requirement, however, yielded drugs that are similar to those already described.

### Strengths and Limitations

We took into account global health status and co-morbidities, but residual confounding cannot be ruled out. Dose-response relationships, carryover effects, and effect of concomitant use of other drugs (including drug-drug interactions) were not considered in this triage strategy. Many new molecular entities are introduced into the market every year and databases that catalog the pharmacology and toxicology of these drugs (including information on molecular targets and gene associations) need to be continually updated. Furthermore, many of these bioinformatics databases may not be publicly available and hence not easily verifiable. Automated filtering and substantiation streamlined the triage and greatly reduced manual work, but full automation is still not possible at this time. Manual verification of the output produced by these workflows, in terms of both accuracy and completeness, remains a crucial step. Finally, safety surveillance for ‘prime suspects’ in electronic healthcare data is, by definition, a hypothesis-generating exercise. Formal clinical and epidemiologic studies to investigate the associations identified by the triage system as necessitating follow-up are obvious and necessary next steps.

## Conclusions

We have proposed a strategy to identify potentially drug-induced acute myocardial infarction using electronic healthcare records that takes into account not only statistical association, but also public health relevance, novelty, and biological plausibility. Although this strategy needs to be further evaluated using other healthcare data sources, the list of ‘prime suspects’ makes a good starting point for further clinical, laboratory, and epidemiologic investigation.

## Supporting Information

Figure S1 **Iterative process of harmonising event definitions and queries across the different databases in EU-ADR.**
(DOC)

Figure S2 **EU-ADR web platform set up.**
(DOC)

Appendix S1 **Description of Longitudinal Gamma Poisson Shrinker (LGPS).**
(DOC)
