# Correction: Drug-Induced Acute Myocardial Infarction: Identifying ‘Prime Suspects’ from Electronic Healthcare Records-Based Surveillance System

**DOI:** 10.1371/annotation/8824e161-bf8f-4f78-81e0-a62a1540276d

**Published:** 2013-09-23

**Authors:** Preciosa M. Coloma, Martijn J. Schuemie, Gianluca Trifirò, Laura Furlong, Erik van Mulligen, Anna Bauer-Mehren, Paul Avillach, Jan Kors, Ferran Sanz, Jordi Mestres, José Luis Oliveira, Scott Boyer, Ernst Ahlberg Helgee, Mariam Molokhia, Justin Matthews, David Prieto-Merino, Rosa Gini, Ron Herings, Giampiero Mazzaglia, Gino Picelli, Lorenza Scotti, Lars Pedersen, Johan van der Lei, Miriam Sturkenboom, on behalf of the EU-ADR consortium

The sentence "M. Molokhia has received support from the National Institute for Health Research (NIHR) Biomedical Research Centre at Guy’s and St Thomas’ NHS Foundation Trust and King’s College London." should not have been part of the Competing Interests section. It should be part of the Acknowledgements. 

