# Supplementary material for: Drug-Induced Acute Myocardial Infarction: Identifying ‘Prime Suspects’ from Electronic Healthcare Records-Based Surveillance System
Source: PLoS One. 2013 Aug 28;8(8):e72148. doi: 10.1371/journal.pone.0072148 (PMC3756064; doi:10.1371/journal.pone.0072148)

**Supplementary Figure S1**. Iterative process of harmonising event definitions and queries across the different databases in EU-ADR


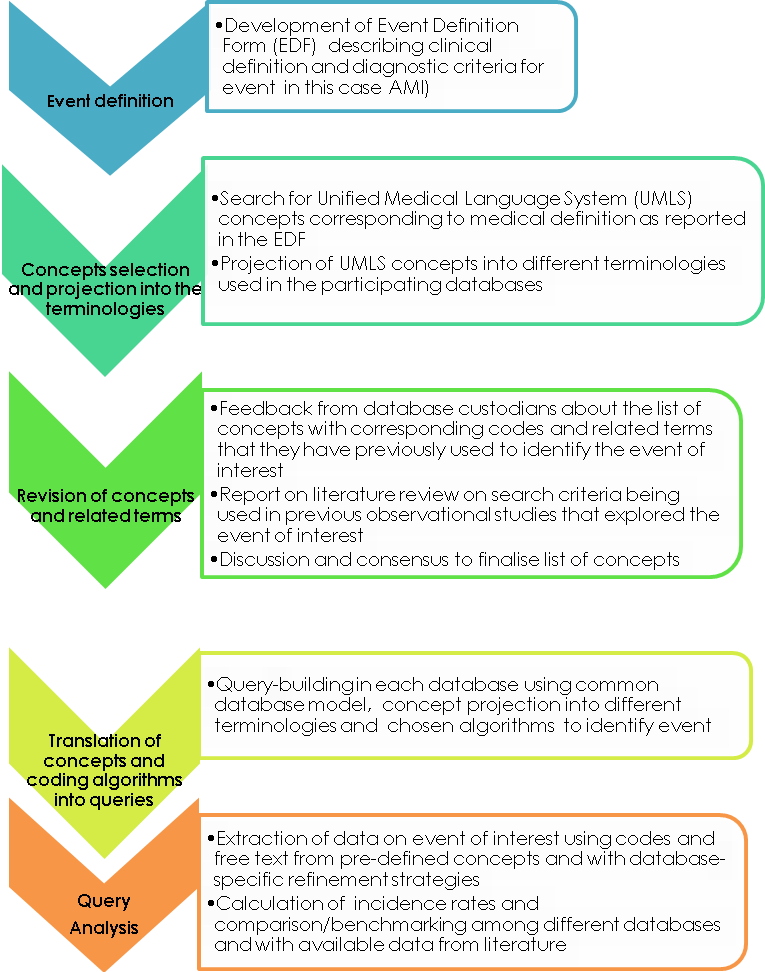

Supplement: Figure S1 — Iterative process of harmonising event definitions and queries across the different databases in EU-ADR. (DOC) [file pone.0072148.s001.doc]
