# Supplementary material for: Drug-Induced Acute Myocardial Infarction: Identifying ‘Prime Suspects’ from Electronic Healthcare Records-Based Surveillance System
Source: PLoS One. 2013 Aug 28;8(8):e72148. doi: 10.1371/journal.pone.0072148 (PMC3756064; doi:10.1371/journal.pone.0072148)
Supplement: Figure S2 — EU-ADR web platform set up. (DOC) [file pone.0072148.s002.doc]

**Supplementary Figure S2**. EU-ADR web platform set up

The EU-ADR web platform has an invite-based registration system that allows authorised researchers to upload and analyse drug-adverse event associations. The web platform integrates several distributed software, streamlined into a single computational workflow Taverna, an open source workflow management system. Both signal filtering (via the biomedical databases Medline, DrugBank and DailyMed) and substantiation (via various protein/gene/pathway databases) are carried out using dedicated bioinformatics methods (<https://bioinformatics.ua.pt/euadr>).


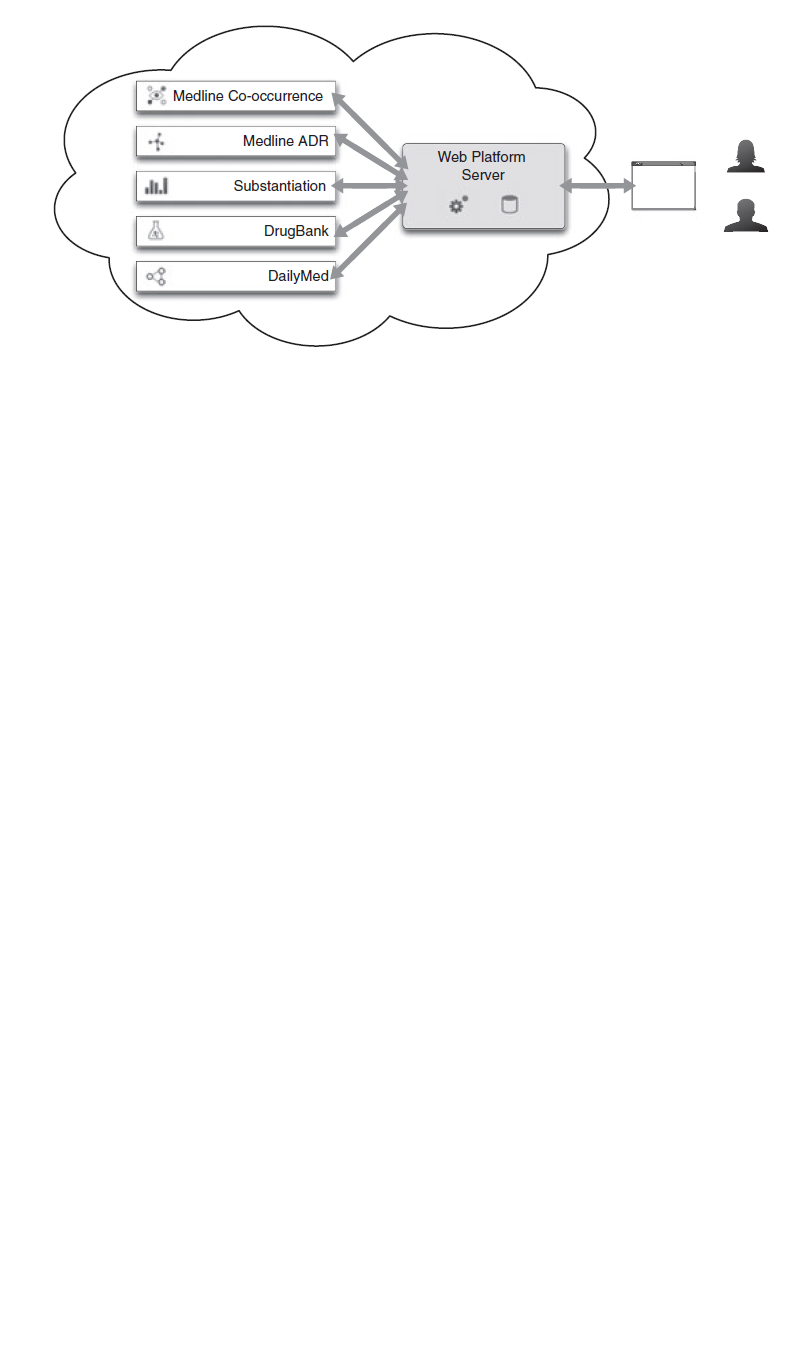


Figure reproduced with permission from: Oliveira JL, Lopes P, Nunes T, Campos D, Boyer S, et al. (2013) The EU-ADR Web Platform: delivering advanced pharmacovigilance tools. *Pharmacoepidemiol Drug Saf 22: 459-467*
