# Supplementary material for: Drug-Induced Acute Myocardial Infarction: Identifying ‘Prime Suspects’ from Electronic Healthcare Records-Based Surveillance System
Source: PLoS One. 2013 Aug 28;8(8):e72148. doi: 10.1371/journal.pone.0072148 (PMC3756064; doi:10.1371/journal.pone.0072148)
Supplement: Appendix S1 — Description of Longitudinal Gamma Poisson Shrinker (LGPS). (DOC) [file pone.0072148.s003.doc]

**Supplementary Appendix S1.** Description of Longitudinal Gamma Poisson Shrinker (LGPS)

LGPS is an adaptation of the Gamma Poisson Shrinker (GPS), an empirical Bayesian method for identifying unusually frequent counts in a large set of spontaneous reports.[[1]](#footnote-2) For every combination of drug X and event A, a table W of report counts is constructed as follows:

|  | Event A | **Not** Event A |
| --- | --- | --- |
| Drug X | W_00_ | W_01_ |
| **Not** Drug X | W_10_ | W_11_ |

Based on W, the baseline (expected) count E for w_00_ is calculated as:

assuming that the probability of an event occurring during exposure is equal to the probability during nonexposure.

LGPS, on the other hand, utilises the exposure information in longitudinal databases and counts patient exposure (in days) instead of reports. The number of events occurring during non-exposed days is used to estimate the expected number of events occurring during exposure:

where t_1_ is the time patients are exposed to drug X, t_0_ is the time patients were included in the database but were not exposed to drug X, w_01_ is the number of distinct events A occurring while not exposed to drug X.

The R package for calculating GPS developed by Ahmed et al. was adapted to calculate the LGPS.[[2]](#footnote-3)

1. DuMouchel W. (1999) Bayesian data mining in large frequency tables, with an application to the FDA spontaneous reporting system. *The American Statistician 53: 190–196*. [↑](#footnote-ref-2)
2. Ahmed I, Haramburu F, Fourrier-Reglat A, Thiessard F, Kreft-Jais C, et al. (2009) Bayesian pharmacovigilance signal detection methods revisited in a multiple comparison setting. *Stat Med 28: 1774-1792*. [↑](#footnote-ref-3)
